# Flexible Aerogels for Thermal Insulation: Fabrication and Application

**DOI:** 10.1021/acsami.5c12107

**Published:** 2025-09-30

**Authors:** Peijie Lin, Xinlin Qing, Qijian Liu, Yuanyuan Yang

**Affiliations:** School of Aerospace Engineering, 12466 Xiamen University, Xiamen 361000, China

**Keywords:** flexible aerogels, thermal insulation, porous materials

## Abstract

Aerogels, known for their lightweight, porous structure and exceptional thermal insulation properties, have attracted increasing attention across research and engineering domains. Recent advancements in material composition and structural design have led to the development of flexible aerogels, broadening their applications across diverse fields. This review provides a comprehensive overview of the fabrication methods for flexible thermal insulation aerogels, including freeze-drying, phase separation, 3D printing, and fiber formation. Their multifunctional applications in aerospace, construction, and battery industry are also highlighted. Recent progress has demonstrated their applicability as lightweight insulation for spacecraft, energy-efficient building materials, and thermal management layers in advanced batteries, underscoring their versatility in both traditional and emerging technologies. Finally, the challenges and future development for flexible thermal insulation aerogels are discussed, offering valuable insights for advancing this promising material.

## Graphical abstract

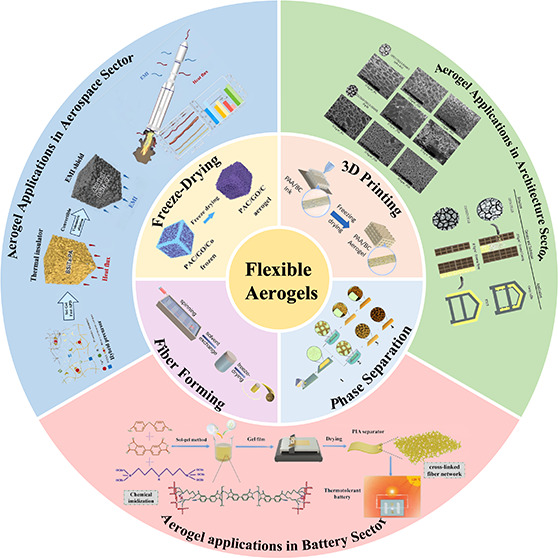

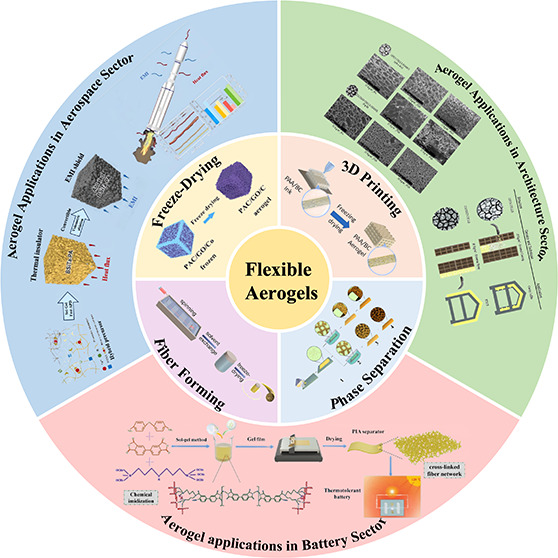

## 1 Introduction

Aerogels, as porous materials with significant scientific and engineering value,^1,2^ have attracted increasing attention. The unique highly porous nanostructure of aerogels endows them with extremely low density and excellent thermal and electrical insulation properties.^3,4^ Since Samuel Stephens Kistler successfully synthesized the first aerogel using supercritical drying in the 1930s, aerogels have emerged as a prominent material in the field of materials science.^5^ With the advancement of research, the aerogel family has expanded, leading to the development of various types and fabrication methods. The definition of aerogels has also broadened to encompass three-dimensional porous materials with gas as the dispersing medium. Their nanostructured frameworks and pore sizes can be finely tuned, enabling unique properties such as low density, high porosity, low thermal conductivity, and large surface area.^6–8^ Due to these exceptional properties, aerogels hold immense potential for applications across multiple domains, including construction,^9,10^ environmental protection,^11,12^ life sciences,^13,14^ aerospace,^15–17^ energy storage,^18–20^ intelligent sensing^21,22^ and chemistry^23–25^ as well as thermal insulation.^26–30^

Despite their remarkable thermal insulation properties, traditional aerogels often exhibit poor mechanical performance due to their inherently fragile nanoporous network and ultralight structure.^31,32^ For instance, silica aerogels are known for their exceptional lightness, high porosity, and outstanding thermal insulation properties. However, they are limited to short-term usage at high temperatures, encountering challenges such as brittleness, susceptibility to breakage, and alterations like shrinkage and deformation during processing.^33–36^ Their mechanical fragility, typically resulting in brittle failure, poses challenges in maintaining structural integrity under practical conditions, particularly in environments requiring resistance to bending or compression.^37–39^ Consequently, this limitation has impeded the broader application of aerogels in engineering domains where mechanical robustness is crucial.^40,41^ Addressing these challenges is vital for unlocking the full potential of aerogels in various applications.^42^

To overcome these limitations, recent research has increasingly focused on the development of flexible thermal insulation aerogels. Efforts in this area have concentrated on designing and fabricating aerogels that incorporate flexible organic or inorganic components or employ specialized microstructural designs to enhance the mechanical strength and resilience of the material. These innovative flexible aerogels not only preserve the exceptional thermal insulation properties characteristic of traditional aerogels but also exhibit enhanced compressibility and recoverability. Such advancements allow them to maintain functionality even under extreme conditions, thereby significantly broadening their potential applications in various sectors.^43–45^ Recent reviews have summarized the development of aerogels from broader perspectives, such as structural design strategies and multifunctional applications,^46,47^ whereas the present review concentrates specifically on flexible thermal insulation aerogels.

This review provides a comprehensive summary of the prevalent fabrication techniques for flexible thermal insulation aerogels, including freeze-drying, phase separation, 3D printing, and fiber-forming methods. The principles of these techniques, along with the resulting material properties and key characteristics of the resulting aerogels, are systematically discussed. Additionally, this review explores the diverse applications of flexible aerogels and highlights the prospects and challenges for advancing this rapidly evolving field (Figure 1).

**Figure 1:**
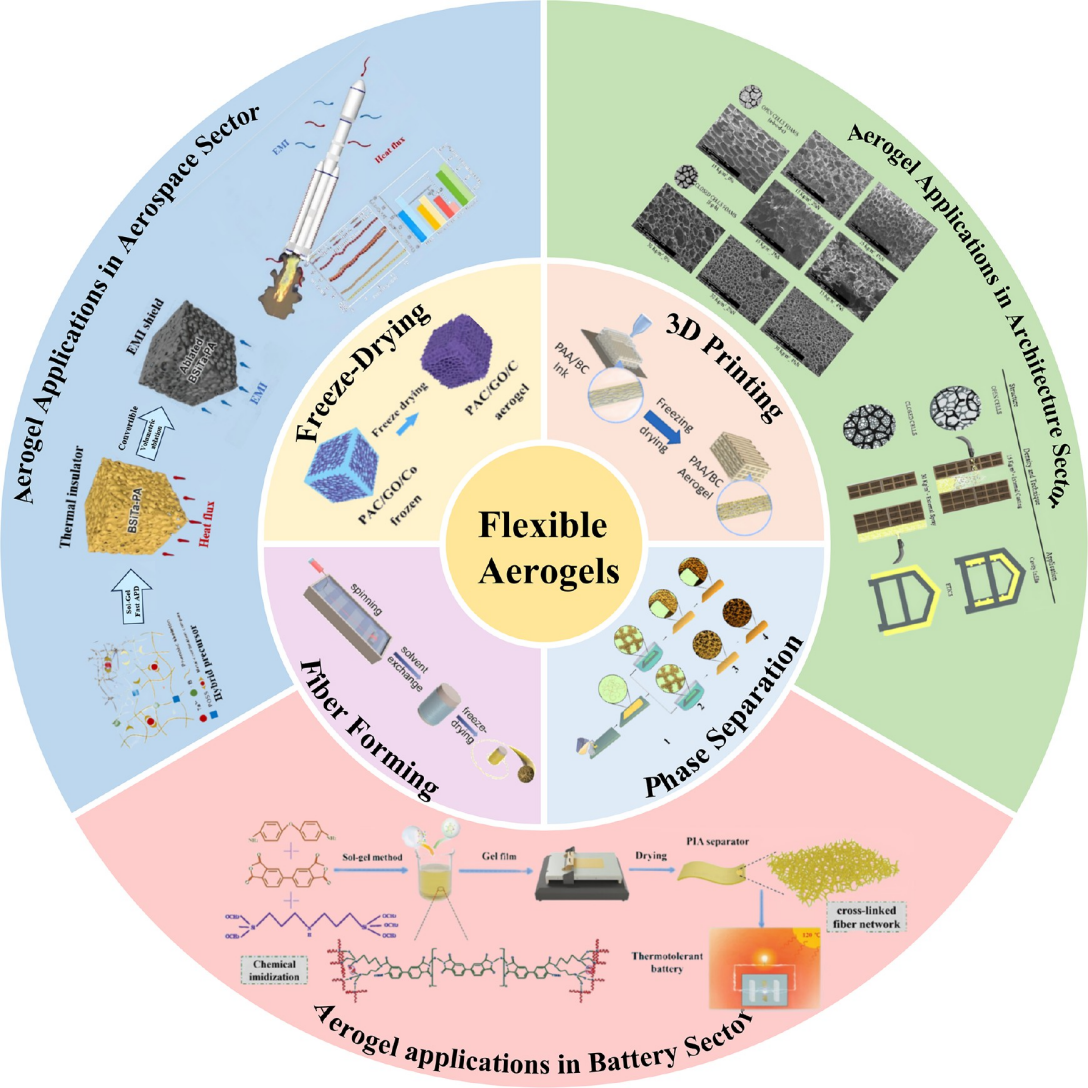
Fabrication techniques and applications of flexible thermal insulation aerogels. Reproduced with permission from reference.^48^ Copyright 2019 Elsevier B.V. Reproduced with permission from reference.^49^ Copyright 2023 Elsevier Ltd. Reproduced with permission from reference.^50^ Available under a CC-BY-NC-ND license. Copyright 2022 Ma et al. Reproduced with permission from reference.^51^ Copyright 2019 American Chemical Society. Reproduced with permission from reference.^52^ Copyright 2018 Elsevier Ltd. Reproduced with permission from reference.^53^ Copyright 2021 Wiley-VCH GmbH.

## 2 Fabrication Techniques of Flexible Aerogels

The flexibility of macroscopic materials is intrinsically linked to their microstructure. Recent advancements in aerogel research have focused on designing and manipulating their microstructures to transition from brittle to flexible states, marking a significant research trend in the field.^54–56^ Various fabrication techniques have been developed to impart flexibility into aerogels. Among them, freeze-drying and phase separation primarily regulate the microstructure, while 3D printing and fiber-forming are mainly approaches for constructing macroscopic architectures. In this section, microstructure-oriented methods are presented first (freeze-drying and phase separation), followed by macroscopic shaping methods (3D printing and fiber-forming).

### 2.1 Freeze-Drying Fabrication Method

Freeze-drying, also known as the ice-segregation-induced self-assembly (ISISA) process,^57^ is a sublimation-based drying technique widely used in the preparation of flexible thermal insulation aerogels. In this process, the material is rapidly frozen at low temperatures, and under vacuum conditions, the frozen water molecules sublimate directly into vapor. This method maintains the structural integrity of the material, avoiding issues such as oxidation or foaming caused by water vapor.

To prepare flexible aerogels, the solution is typically frozen in a cryogenic bath and subjected to vacuum sublimation in a freeze-dryer, resulting in a porous structure.^58–60^ For instance, Zhang et al. fabricated aerogels by freeze-drying a mixture of graphene oxide (GO), polyimide (PI) precursor, and cobalt acetate, followed by thermal annealing in a nitrogen atmosphere as Figure 2a. The resulting aerogels exhibited excellent compressive strength, tensile resilience, and flexibility.^48^ Similarly, Liang et al. synthesized silicon carbide (SiC) nanowire aerogels using resorcinol, silicon dioxide (SiO_2_) sol, and formaldehyde as precursors. After freeze-drying and high-temperature sintering in an argon (Ar) atmosphere, the aerogels demonstrated ultrahigh porosity, low thermal conductivity, and good flexibility (Figure 2b).^61^

**Figure 2:**
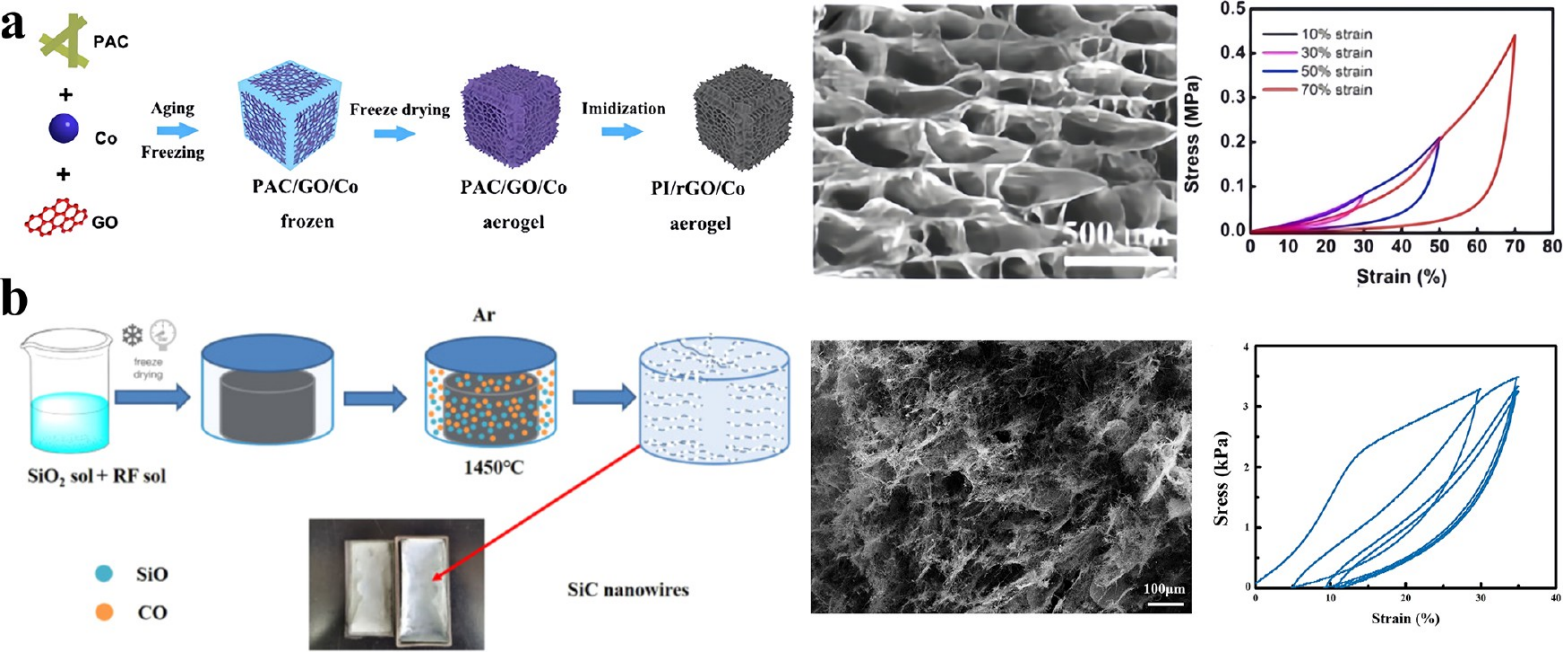
Preparation and properties of freeze-drying aerogel materials. (a) Fabrication methods, microstructure, and mechanical properties of flexible PI aerogel. Reproduced with permission from reference.^48^ Copyright 2019 Elsevier B.V. (b) Preparation, microstructure, and mechanical properties of flexible SiC nanowire aerogel. Reproduced with permission from reference.^61^ Copyright 2022 Elsevier Ltd. and Techna Group S.r.l.

Aerogels prepared using conventional freeze-drying methods typically feature a disordered porous structure, which limits their ability to efficiently transfer stress and heat, thereby constraining their application potential. Directional freeze-drying, an advancement of the conventional method, addresses this limitation by controlling the growth direction of ice crystals during freezing, enabling the fabrication of flexible aerogels with oriented porous microstructures.^62,63^ This technique used a specific cryogen and a temperature gradient to guide ice crystal growth along a desired direction.

By adjusting freezing parameters—such as temperature, freezing agents, and methods—allows precise control of the ice crystal structure. Following the freezing process, the gel is demolded and subjected to freeze-drying, resulting in aerogels with ordered and tunable structures.^64^ For example, Xu et al. synthesized anisotropic PI aerogels using a mixture of polyamic acid (PAA) precursors, which were cast into molds, subjected to unidirectional freezing, and vacuum freeze-dried (Figure 3a). Subsequent thermal imidization produced aerogels with anisotropic microstructures and excellent mechanical properties.^65^ Similarly, Dou et al. utilized a fiber dispersion subjected to multidirectional freezing, promoting rapid ice crystal growth and forming a three-dimensional nanofiber network (Figure 3b). Freeze-drying and calcination produced ceramic nanofiber aerogels with exceptional thermal stability, elasticity, and fatigue tolerance, even under high compression and buckling strain.^66^

**Figure 3:**
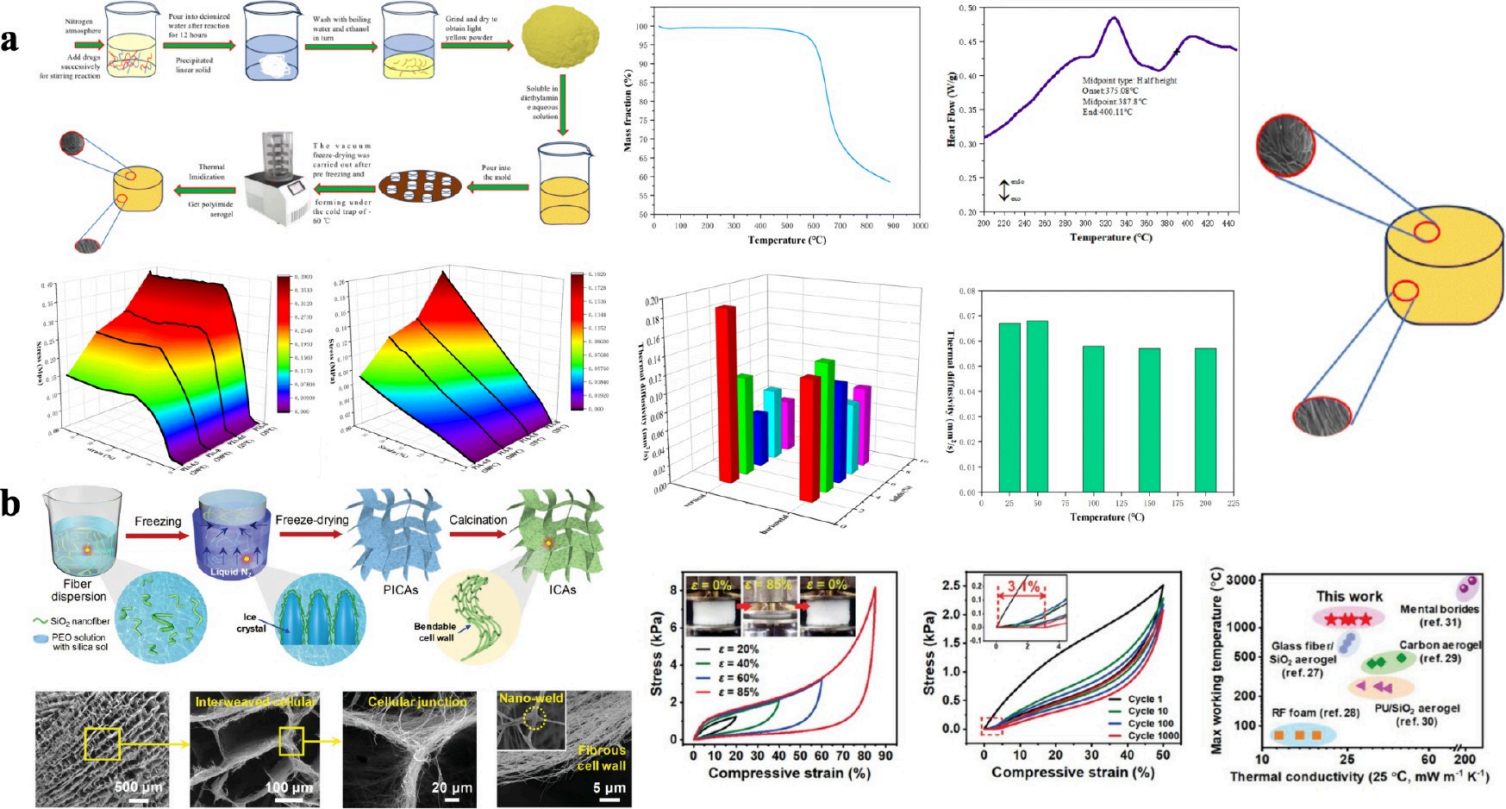
Preparation and properties of directional freeze-dried aerogel materials. (a) Freeze-drying methods, microstructure, mechanical properties and thermal properties of anisotropic PI aerogels. Reproduced with permission from reference.^65^ Available under a CC-BY-NC 3.0 license. Copyright 2024 Xu et al. (b) Freeze-drying methods, microstructure, mechanical properties and thermal stability of nanofibrous aerogels. Reproduced with permission from reference.^66^ Copyright 2020 Wiley-VCH GmbH.

In summary, freeze-drying methods can effectively preserve porous networks and lead to lightweight aerogels. The resulting flexibility is related to both process control and material choice. While conventional silica-based aerogels remain relatively brittle, polymeric matrices or nanofiber-reinforced systems show superior recoverability. Furthermore, directional freeze-drying introduces anisotropic architectures that efficiently distribute stress, thereby enhancing bending and compression tolerance. These features demonstrate that freeze-drying routes can be tuned not only for thermal insulation but also for tailoring flexibility.

### 2.2 Fabrication of Flexible Thermal Insulation Aerogels via Phase Separation

Phase separation is a commonly used method for fabricating flexible aerogel films,^67^ which is based on the thermodynamic instability of polymer solutions. When conditions such as temperature, solvent composition, or concentration change, the homogeneous solution becomes unstable and separates into two distinct phases: a polymer-rich phase and a polymer-poor phase.^68^ In this process, the polymer-rich phase forms the solid network structure of the aerogel, while the polymer-poor phase primarily consists of the solvent. By controlling the interaction between these two phases and the phase separation process, aerogel films with specific microstructures and properties can be produced. In aerogel manufacturing, thermal induction and nonsolvent induction are two commonly used methods to regulate phase separation behavior.^69^

For thermal induction method, the concentration of the polymer-poor phase and the polymer-rich phase in the solution is significantly influenced by temperature.^70^ When the concentration difference between these two phases reaches a critical value, the phase separation process begins.^71^ For example, Ma et al. employed thermally induced phase separation (TIPS) using a mixture of poly­(methyl methacrylate) (PMMA), ethanol, and water to synthesize robust and lightweight PMMA-modified silica aerogels. The TIPS process involved dissolving PMMA powder completely in a mixture of ethanol and water at 60 °C until the initially turbid mixture became transparent. The prepared gel was then immersed in this solution and allowed to cool to room temperature. After preserving the gel in the PMMA solution at 10 °C for 3 days, supercritical CO_2_ drying was used to obtain the aerogel.^72^ The resulting aerogel exhibited low density, uniform pore size, high porosity, low thermal conductivity, and excellent mechanical properties, as shown in Figure 4a.

**Figure 4:**
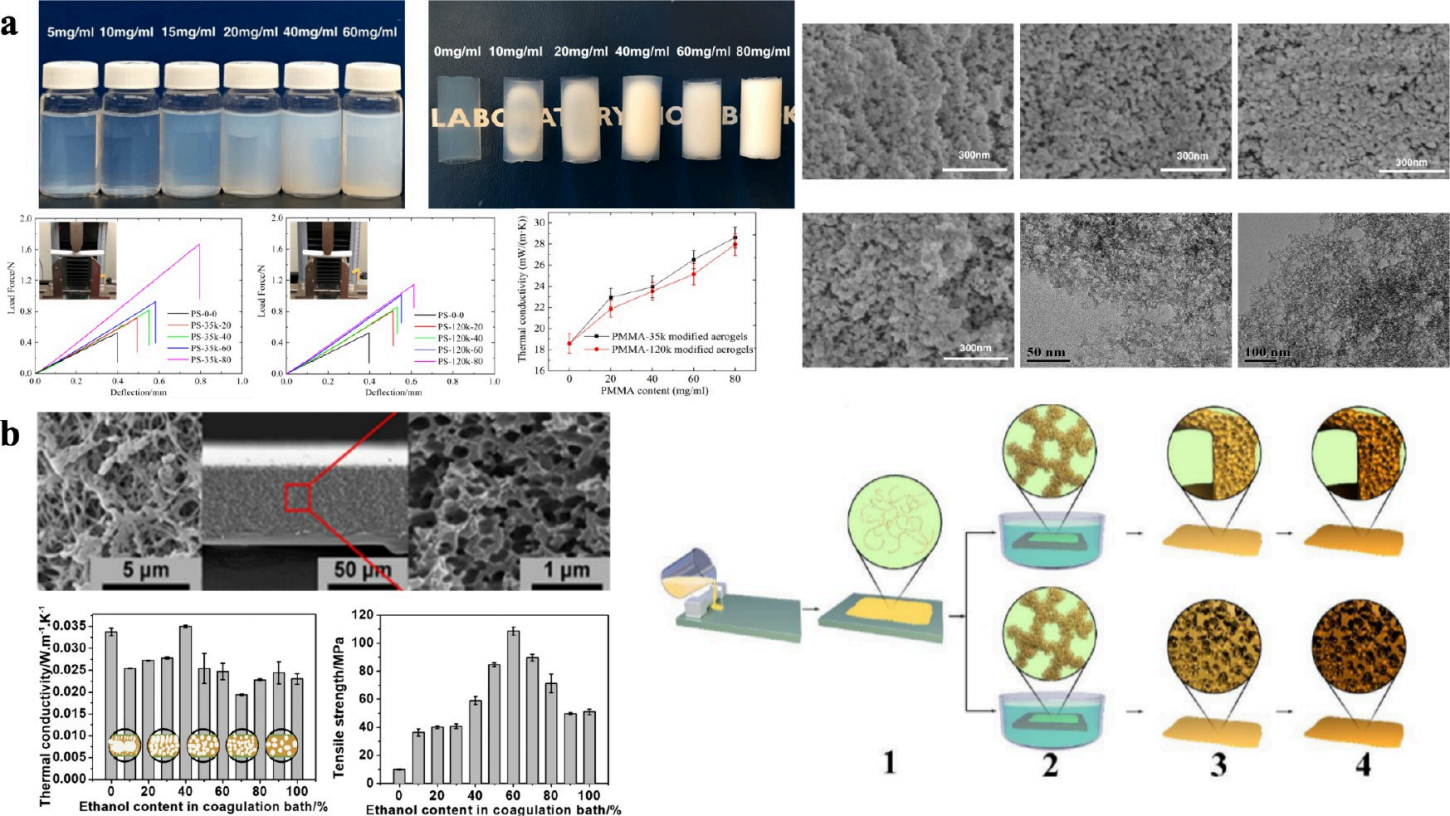
Preparation and properties of phase-separation aerogel materials. (a) Flexible PMMA-modified silica aerogels prepared by thermally induced phase separation. Reproduced with permission from reference.^72^ Available under a CC-BY license. Copyright 2023 Ma et al. (b) Flexible and thermal insulated PI aerogels by nonsolvent induced phase separation. Reproduced with permission from reference.^50^ Available under a CC-BY-NC-ND license. Copyright 2022 Ma et al.

Nonsolvent induced phase separation (NIPS) is another commonly used technique for fabricating polymer films. In this process, the addition of a nonsolvent plays a crucial role in triggering phase separation, regulating the film structure and its properties.^73^ When a polymer solution (typically containing polymer, solvent, and possibly additives) comes into contact with a nonsolvent, the poor solubility of the nonsolvent for the polymer affects the interaction between polymer and solvent molecules. This results in an increase in the concentration difference between the polymer-poor phase and the polymer-rich phase in the solution, thereby lowering the critical value for phase separation and initiating the phase separation process.^74^ Ma et al. prepared flexible PI aerogel films using a simple nonsolvent-induced phase separation method combined with room-temperature drying, as shown in Figure 4b.^50^ By immersing the composite film in a nonsolvent mixture, phase separation was induced, resulting in a wet porous film. The final flexible PI aerogel film was achieved by heating the film in an argon atmosphere, exhibiting a low thermal conductivity and a high tensile strength.

In general, phase separation methods are effective for producing flexible aerogel films with tunable mechanical properties. The degree of flexibility is governed mainly by the choice of polymer matrix. This dependence on the base material highlights the central role of matrix selection in achieving targeted flexibility.

### 2.3 Fabrication of Flexible Thermal Insulation Aerogels via 3D Printing

Polymer aerogels, widely used in thermal insulation, offer superior mechanical properties compared to conventional silica aerogels.^75–77^ However, polymer aerogels are commonly produced through the sol–gel process,^78^ resulting in aerogels with simple structures such as films, particles, or monoliths, limiting their application in advanced designs.^79,80^ In recent years, the emerging technology of 3D printing has addressed this limitation.^81^ Combining 3D printing technology with aerogel fabrication allows for the production of aerogels with complex microstructures and intricate shapes, offering approaches to the structural design of flexible thermal insulation aerogels.^82,83^ Existing 3D printing methods for aerogels can be broadly categorized into direct-ink-writing (DIW) 3D printing method and template-assisted 3D printing method.

DIW 3D printing method involves programmatically controlling the extrusion of a suitable ink to deposit complex 3D microstructures.^84^ Typically, the process begins with preparing the ink, which is formulated from various molecules, polymers, or particulate substances^85^ to ensure appropriate rheological properties (such as viscosity and flowability) for extrusion through a nozzle. Parameters such as printing path, speed, layer thickness, and extrusion pressure are then set. The ink is extruded through a controllable nozzle and deposited along the predetermined path on a substrate. The printhead builds the 3D structure layer by layer according to the designed model. The choice of parameters depends on the ink characteristics and the desired microstructure design. After printing, the structure usually undergoes curing and drying. This method is known for its high precision, material versatility, and cost-effectiveness. For example, Wang et al. fabricated flexible thermal insulation SiO_2_ aerogels using DIW. A tailored ink comprising water, poly vinyl alcohol (PVA) solution, Tween 80, hydroxypropyl methylcellulose, and silica was used to print various 3D models (Figure 5a).^86^ The printed samples underwent surface treatment and a two-stage solvent exchange, resulting in SiO_2_ aerogels with low thermal conductivity and excellent insulation properties at medium to high temperatures.

**Figure 5:**
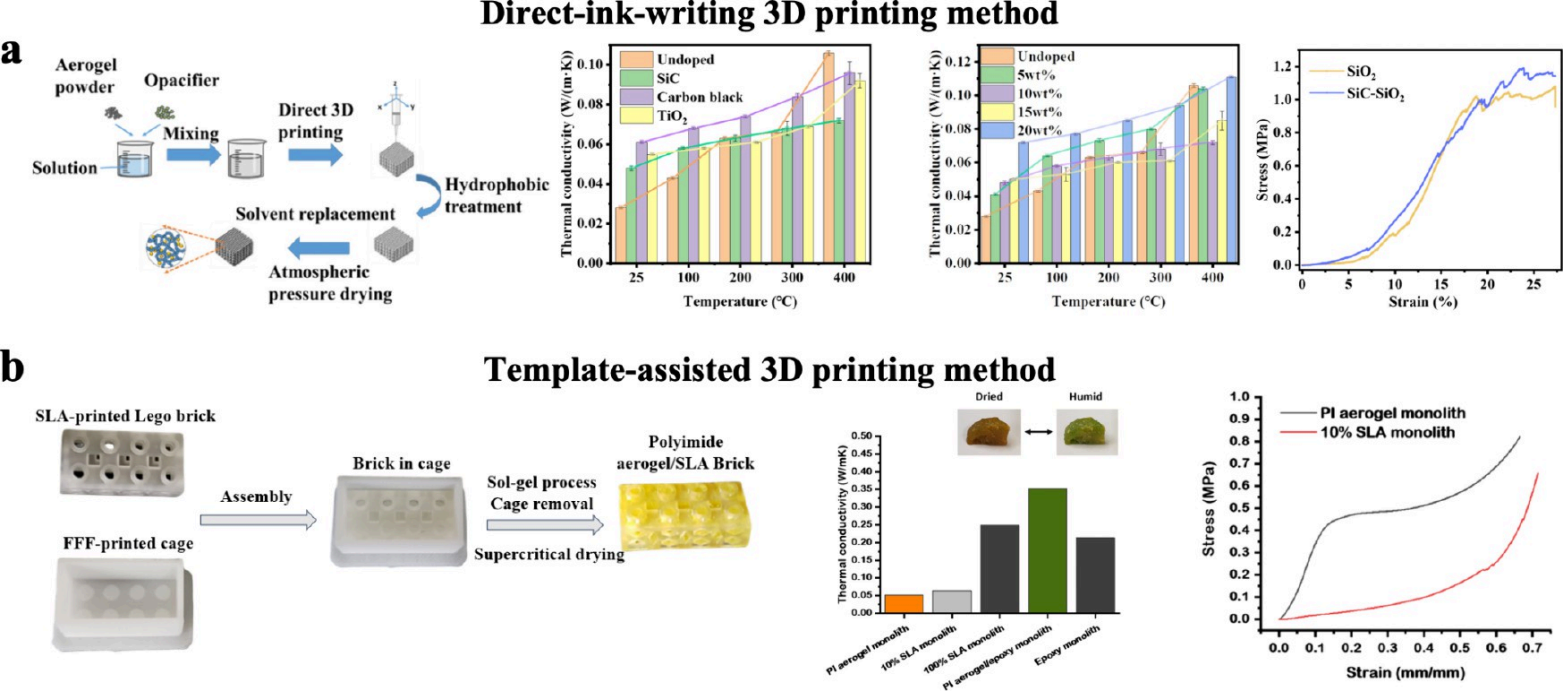
Preparation and properties of 3D printing aerogel materials. (a) Flexible thermal insulation SiO_2_ aerogels prepared by a DIW 3D printing method. Reproduced with permission from reference.^86^ Copyright 2023 Elsevier B.V. (b) Modular aerogel/acrylate bricks prepared by a template-assisted 3D printing method. Reproduced with permission from reference.^91^ Copyright 2021 Elsevier B.V.

Template-assisted 3D printing is another manufacturing technique for designing aerogel microstructures. This method begins with designing a mold based on the desired aerogel structure, using materials that are soluble in a specific solvent but insoluble in the solvents used for aerogel preparation, allowing for easy mold removal. Such molds are often referenceerred to as sacrificial molds.^87–90^ The aerogel precursor, such as a sol–gel or polymer solution, is then filled into the mold’s cavities, where it undergoes curing to solidify within the mold. Subsequent drying processes, such as solvent exchange and supercritical drying, the aerogel’s microstructure is created. Finally, the mold material is dissolved or removed, yielding aerogels with complex microstructures as designed. Yao et al. introduced a template-assisted 3D printing technique to produce modular aerogel/acrylate bricks suitable for thermal insulation applications, as shown in Figure 5b.^91^ The preparation process involves designing and 3D printing the molds, and then injecting the aerogel precursor solution into them. After the cross-linking reactions of gels completed, the molds are release by soaking in solvents. Finally, the aerogel undergoes supercritical drying to produce modular PI and acrylate aerogel bricks. The resulting aerogels feature relatively low density, low thermal conductivity, customizable structures, and excellent mechanical properties.

Freeze-drying, solvent exchange, and supercritical drying are widely adopted postprocessing method following the printing step.^86,91,92^ This process effectively preserves both the macroscopic shape and the microscopic porosity of the aerogels, providing the material with excellent thermal insulation properties and flexibility. For example, Ma et al. utilized high-aspect-ratio bacterial cellulose (BC) as a rheological modifier to formulate a water-based ink with low solid content but high yield stress, enabling the printing of PI/BC aerogels with various macroscopic structures.^49^ After printing, the samples were freeze-dried to remove moisture, then thermally imidized to obtain PI/BC composite aerogels. The resulting aerogels exhibited relatively low density, low thermal conductivity, and excellent mechanical properties.

Overall, 3D printing provides not only geometric freedom but also the opportunity to engineer flexibility through architecture design and ink composition. The structure design can improve resistance to bending and cyclic loading, while additives such as polymeric and nanocellulose can further enhance ductility. Compared with bulk aerogels, 3D-printed aerogels can combine structural complexity with mechanical resilience, making them a promising pathway to flexible aerogel design.

### 2.4 Fabrication of Flexible Thermal Insulation Aerogels via Fiber Forming

Aerogels traditionally appear in three-dimensional blocks, particles, or two-dimensional films. Recently, a new form of aerogel, aerogel fibers, has emerged. This fiberization process transforms conventional aerogels into fine submicron or nanometer-scale fibers, potentially offering significant performance enhancements.^93–95^ Aerogel fibers exhibit improved mechanical strength, better flexibility, higher tensile strength, and greater ductility. These attributes make them highly suitable for applications requiring high mechanical adaptability and elasticity. They are commonly produced by drawing a sol into fiber form through specific processes followed by gelation. Although aerogel fibers exhibit reduced mechanical strength compared to traditional fibers, they possess exceptional flexibility and thermal insulation properties, consistent with the inherent characteristics of aerogels. The three primary methods for producing flexible aerogel fibers with uniform porous structures are wet spinning, freeze spinning, and sol–gel-based phase transition strategies.

The wet spinning process, also known as wet spinning, is a commonly used method for manufacturing aerogel fibers. This process involves extruding a polymer solution through a spinneret into a coagulation bath to form fibers. For aerogel fiber production, solutions containing aerogel precursors are used, which undergo phase separation during wet spinning to create wet fiber gels. Subsequently, appropriate drying techniques, such as supercritical fluid drying or atmospheric pressure drying, are employed to remove the solvent, developing aerogel fibers with a porous structure. Postprocessing treatments, such as hydrophobic modification^96^ and chemical reduction,^97^ can further functionalize the resulting aerogel fibers. For example, Liu et al. proposed a fabrication method for aerogel fibers by extruding the Kevlar nanofibrous (KNF) dispersions from a pump-controlled syringe into a coagulation bath. The gel fibers were then immersed in a tert-butanol aqueous solution, followed by freeze-drying. Afterward, the fibers underwent chemical nickel plating and were immersed in molten polyethylene glycol, then baked to obtain KNF aerogel fibers, as shown in Figure 6a.^51^ The resulting aerogel fibers exhibited a high specific surface area and broad thermal stability. The flexible and robust KNF aerogel fibers were then woven into textiles, demonstrating excellent thermal insulation performance at extreme temperatures.

**Figure 6:**
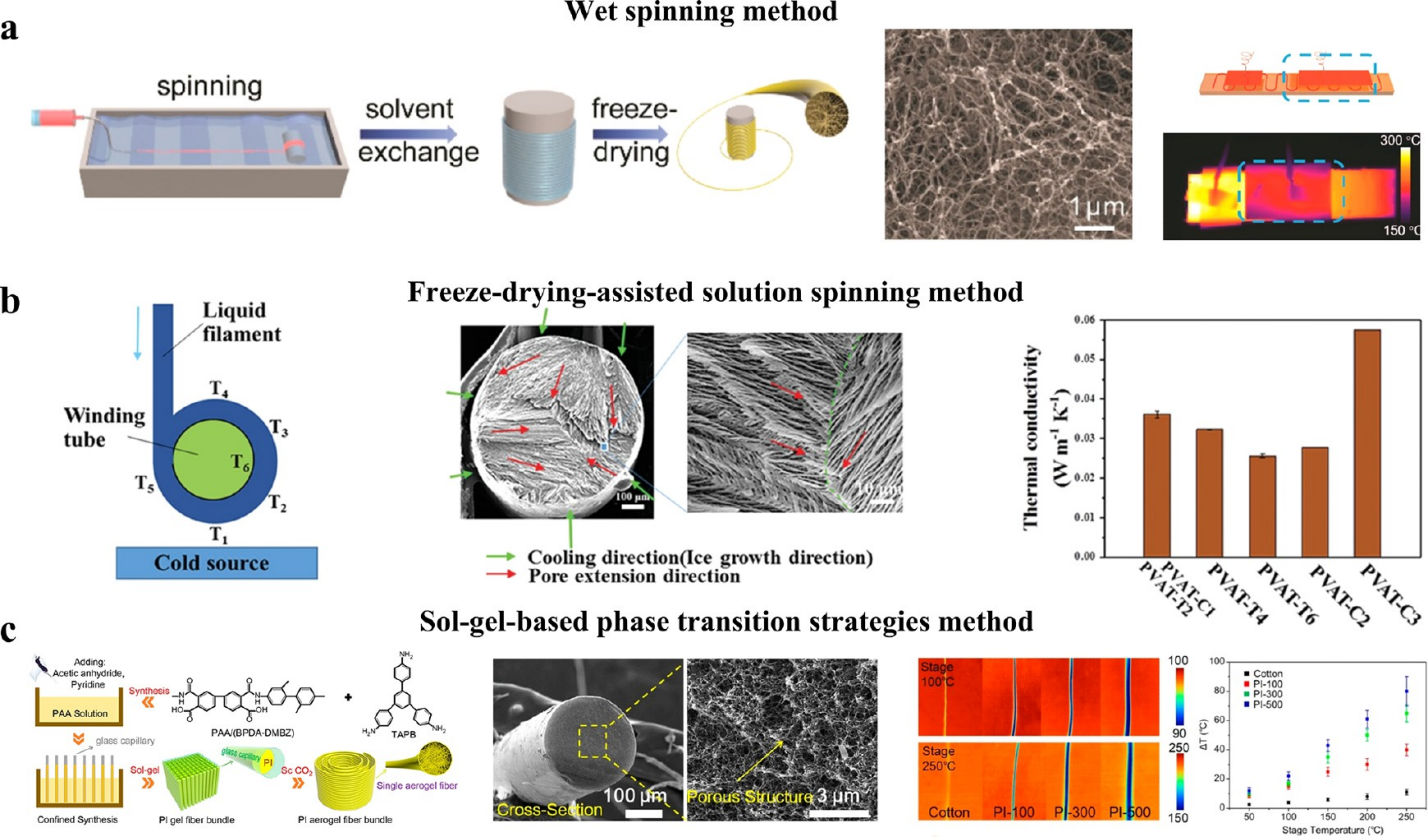
Preparation and properties of flexible aerogel fibers. (a) KNF aerogel prepared by a wet spinning method. Reproduced with permission from reference.^51^ Copyright 2019 American Chemical Society. (b) PVA aerogel fibers with an aligned porous morphology prepared by a freeze-drying assisted solution spinning method. Reproduced with permission from reference.^99^ Copyright 2021 Wiley-VCH GmbH. (c) PI aerogel fibers prepared by a sol–gel-based phase transition strategies method. Reproduced with permission from reference.^100^ Copyright 2021 American Chemical Society.

Freezing spinning, also known as freeze-drying spinning or cryogenic spinning, is another technique for fabricating flexible insulating aerogel fibers. In this process, the spinning solution is extruded from a syringe pump into a cryogenic environment, such as liquid nitrogen, where it rapidly solidifies into ice crystals. By adjusting the extrusion speed and the temperature of the cooling source, the fibers can be frozen at a controlled rate, determining their final structure. Once the fibers are formed, they are collected and subjected to further freeze-drying to remove the ice crystals. This method enables the creation of uniform and continuous porous structures within the fibers, resulting in aerogel fibers with good thermal insulation properties. The microscopic structure within the fibers, which significantly impacts their mechanical and insulating properties, could be controlled by adjusting parameters such as solution concentration, viscosity, extrusion speed, and freezing temperature. Based on that, aerogel fibers with unique porous structures and superior insulating characteristics can be synthesized.^98^ Dai et al. prepared the PVA aerogel fibers by placing the PVA solution in a cooling chamber equipped with a low-temperature cooling pump. The solution underwent a freeze–thaw (FT) process followed by thawing, then loaded into a spinning tube for freeze spinning. The fibers were collected on a spool and simultaneously frozen with liquid nitrogen beneath the spool. After that, the frozen fibers were stored in a freezer then vacuum freeze-dried to obtain PVA aerogel fibers, as shown in Figure 6b.^99^ The resulting aerogel fibers exhibited excellent weaving capability, superior mechanical properties, and outstanding thermal insulation.

The Sol–Gel Confined Transition (SGCT) method is an innovative approach for fabricating high-performance flexible thermal insulation aerogel fibers. This technique involves mixing metal–organic compounds or metal salts with a solvent and then immersing a mold with a designed capillary structure into the solution. Driven by surface tension, the solution penetrates the capillary tubes, where hydrolysis and condensation reactions are confined within the capillaries. This ensures the formation of wet gel fibers with controlled morphology. The resulting wet gel fibers are then subjected to aging, washing, solvent exchange, and drying processes to yield aerogel fibers with a porous structure. This method addresses the conflict between the static sol–gel transition and the dynamic spinning process that often encountered in traditional wet-spinning techniques. For instance, Li et al. employed a sol–gel confined transformation strategy to fabricate PI aerogel fibers with a mesoporous structure. The process involved using surface tension to drive the PI aerogel precursor solution into capillary molds, followed by a static sol–gel process in a sealed environment to form gel fibers. The fibers were then dried using supercritical CO_2_ to achieve a mesoporous structure, as shown in Figure 6c.^100^ The resulting PI aerogel fibers exhibited a high specific surface area, good mechanical properties, outstanding hydrophobicity, and remarkable flexibility. The aerogel textiles made from these PI aerogel fibers demonstrated superior thermal insulation performance across a wide temperature range, even under harsh environmental conditions.

These fiber-forming strategies naturally enhance aerogel flexibility due to the intrinsic advantages of fibrous morphology. Aerogel fibers and fabrics show improved tensile strength, bending resistance, and fatigue tolerance compared with bulk monoliths, enabling them to be woven or assembled into flexible textiles.

In summary, the macroscopic flexibility of aerogels can be controlled through their microstructural design and matrix materials selection. The existing methods for constructing flexible aerogel microstructures primarily include directional freeze-drying, phase separation, 3D printing, and aerogel fibers/fabrics, as outlined in Table 1. While aerogels produced by these methods exhibit commendable thermal insulation and flexibility, challenges remain, such as unclear regulatory mechanisms, high production costs, and lengthy fabrication times. Therefore, the development of improved technologies and innovative approaches—such as bioinspired design concepts,^101^ 4D printing,^102^ and other advanced structural engineering strategies—is essential for further enhancing the overall performance of flexible thermal insulation aerogels.

**Table 1:** Comparison of Flexible Thermal Insulation Aerogels Prepared by Different Methods

Methods	Materials	Mechanical properties	Stability	Thermal conductivity	Reference.
Freeze-drying	PI/rGO/Co aerogels	0.506 MPa compression modulus	1000 cycles	0.040 W/(m·K) at 25 °C and 0.046 W/(m·K) at 100 °C	^48^
Freeze-drying	SiC nanowire aerogels	/	/	0.035 W/(m·K)	^61^
Directional freeze-drying	Anisotropic PI aerogels	Compressive modulus at 200 °C is 1.60 MPa	/	Thermal diffusivity down to 0.057 mm^2^ s^–1^	^65^
Directional freeze-drying	Nanofibrous aerogels	/	100000 cycles	0.0223 W/(m·K)	^66^
DIW 3D Printing	SiO_2_ aerogels	/	/	0.028 W/(m·K)	^86^
Template-assisted 3D printing	Modular aerogel/acrylate bricks	/	/	0.05 mW/(m·K)	^91^
Thermally induced phase separation	Silica Aerogels	6.98–12.6 MPa flexural modulus and 3.22–5.05 MPa compression modulus	/	19.20–28.61 mW/(m·K)	^72^
Nonsolvent induced phase separation	PI aerogels	>0.4 GPa tensile modulus	/	0.019 W/(m·K)	^50^
Wet spinning	KNF Aerogels	/	/	0.037 W/(m·K)	^51^
Freezing spinning	Continuous Poly(Vinyl Alcohol) Aerogel Fibers	/	/	0.026 W/(m·K)	^99^
Sol–gel-based phase transition strategies	PI Aerogel Fibers	123 MPa elastic modulus	/	0.025–0.032 W/(m·K)	^100^

## 3 Applications of Flexible Thermal Insulating Aerogels

Flexible thermal insulating aerogels, known for their extremely low thermal conductivity, low density, compressive resistance, and good flexibility, have seen extensive applications across various fields in recent years. Their remarkable resilience and bending resistance address the limitations of traditional rigid aerogels, while their superior thermal insulation properties and adaptable structural designs unlock significant potential in aerospace, construction, and battery technologies. This section will explore the specific applications of flexible thermal insulating aerogels in these domains.

### 3.1 Thermal Insulating Applications of Flexible Aerogels in Aerospace

In aerospace applications, materials are required to meet exceptionally demanding performance criteria, particularly under high-temperature and extreme environmental conditions. Materials must not only exhibit excellent thermal insulation but also meet rigorous standards for weight, strength, and durability. Conventional insulation materials, such as ceramic fibers and polyurethane foams, often fail to meet these demands. Flexible thermal insulating aerogels, with their unique nanoscale porous structure and exceptionally low thermal conductivity, are increasingly gaining favor in the aerospace industry. Beyond their low thermal conductivity and thermal stability, flexible aerogels can withstand mechanical vibrations, folding, and bending during launch and re-entry, conditions under which conventional brittle aerogels would fracture. This mechanical adaptability makes flexibility a decisive property for aerospace thermal protection and spacesuit insulation.

One prominent application of aerogels lies in spacecraft insulation. These advanced materials address the critical need for lightweight yet highly effective thermal barriers capable of withstanding the extreme conditions encountered during space missions. Their unique combination of properties - including ultralow thermal conductivity, exceptional thermal stability, and remarkable mechanical resilience - makes them particularly suitable for protecting spacecraft components from the intense heat generated during atmospheric re-entry and other high-temperature operations. Recent research has demonstrated the potential of aerogel formulations in this demanding application. For example, Niu et. al developed a boron–silica–tantalum ternary hybrid phenolic aerogel (BSiTa-PA) with exceptional thermal stability, extensive mechanical strength, low thermal conductivity, and heightened ablative resistance (Figure 7).^103^ The development of such aerogel lays the foundation for the advancement of insulating materials in next-generation aerospace applications. Xu et al. synthesized hexagonal boron nitride aerogels (hBNAGs) and β silicon carbide aerogels (βSiCAGs) using a specially designed three-dimensional graphene aerogel template.^104^ These aerogels also demonstrated exceptional thermal stability under rapid thermal shocks and prolonged high-temperature exposure. Even after 1 week of exposure at 1400 °C, the aerogels showed negligible mass and volume loss, making them highly suitable for thermal protection systems in spacecraft operating under extreme conditions.

**Figure 7:**
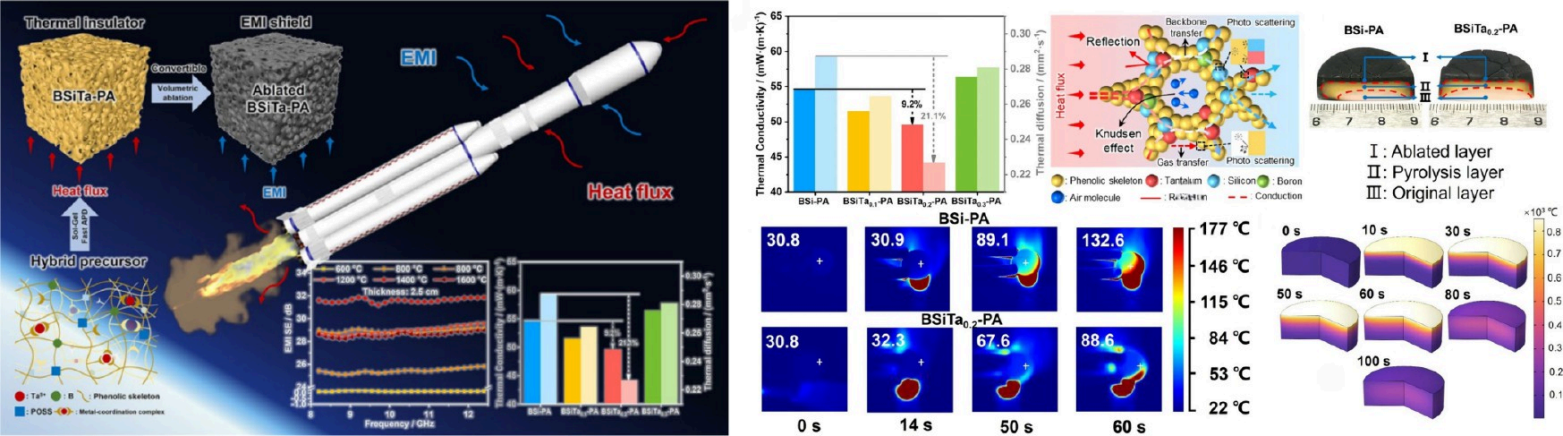
Flexible insulating aerogel in aerospace applications for spacecraft thermal protection. Reproduced with permission from reference.^103^ Available under a CC-BY license. Copyright 2024 Niu et al.

Beyond spacecraft, aerogels also hold significant promise for spacesuit insulation. These specialized applications demand materials that not only provide excellent thermal insulation but also offer flexibility, breathability, and additional safety features such as flame retardancy. The lightweight nature of flexible aerogels is particularly advantageous for spacesuit applications, where minimizing weight while maintaining protection is crucial. Moreover, the adaptability of aerogel formulations allows for the incorporation of various functional additives to enhance specific properties, opening new possibilities for advanced personal protective equipment. Guo et al. developed lightweight flame-retardant cel-lulose nanofiber (CNF) sponge-like aerogels using *N*-methylol dimeth-ylphosphonopropionamide (MDPA) and 1,2,3,4-butanetetracarbo-xylic acid (BTCA) as auxiliary additives.^105^ The resulting aerogels exhibited good flame-retardant properties, outstanding self-extinguishing behavior, a significantly increased char residue, superior thermal insulation, and a low thermal conductivity. The combination of these properties makes CNF-based aerogels promising candidates for thermal protection equipment, particularly in advanced spacesuit applications.

In aerospace applications, flexible thermal insulation aerogels could enhance the reliability and lightweight nature of thermal protection systems for spacecraft, rockets, and aircraft engines. Their extremely low thermal conductivity and superior thermal protection capabilities provide safer and more efficient solutions for space vehicles. Furthermore, their application in spacesuit insulation and aviation equipment protection highlights their potential in extreme environments.

### 3.2 Thermal Insulating Applications of Flexible Aerogels in Architecture

The construction industry is another major application area for thermal insulation aerogels. Residential and commercial buildings are responsible for consuming a sizable share of the global energy consumption. In addition to improving occupant comfort and addressing the growing demand for energy, innovative building materials and technologies offer a safe strategy for geoengineering solutions to mitigate global climate change, making energy efficiency a critical aspect of building design.^106^ As a new class of high-performance insulation material, thermal insulation aerogels are gradually replacing traditional insulation materials in the construction sector due to their superior thermal insulation properties and ease of installation.

The application of aerogels in building envelope insulation represents a transformative approach to improving thermal performance in modern construction. These advanced materials could enhance the energy efficiency of walls, roofs, and floors while overcoming the limitations of conventional insulation solutions. Their ultralow thermal conductivity and thin-profile installation capabilities make them particularly valuable for both new construction and retrofit projects where space constraints often pose challenges. In addition to insulation, flexibility is particularly valuable because aerogel must be cut, folded, and fitted around irregular building surfaces. Flexible aerogels maintain integrity without cracking, improving durability and reducing installation complexity. Recent developments in aerogel-enhanced insulation systems demonstrate their potential to significantly reduce thermal bridging and improve overall building performance. For example, Stazi et al. developed sprayed polyurethane foams for insulation, which can be used either as cavity wall infills or external insulation layers depending on their density (Figure 8).^52^ The incorporation of nanoclay significantly enhanced the performance of the foam, leading to reduced cell diameter, more uniform cellular morphology, lower thermal conductivity, improved moisture sorption and vapor permeability, as well as increased compressive and tensile strength. Aspen Aerogels company developed an aerogel-based insulation material called Spaceloft, which is a flexible, fiber-reinforced silica aerogel blanket. At 0 °C, its thermal conductivity is 0.013 W/mK, which is 2 to 2.5 times lower than traditional insulation materials. This blanket can reduce thermal bridging that often occurs due to studs in wood or steel frame building envelopes.^107^ In general, these aerogel technologies are making a significant difference in building insulation, helping to make buildings more energy-efficient and comfortable.

**Figure 8:**
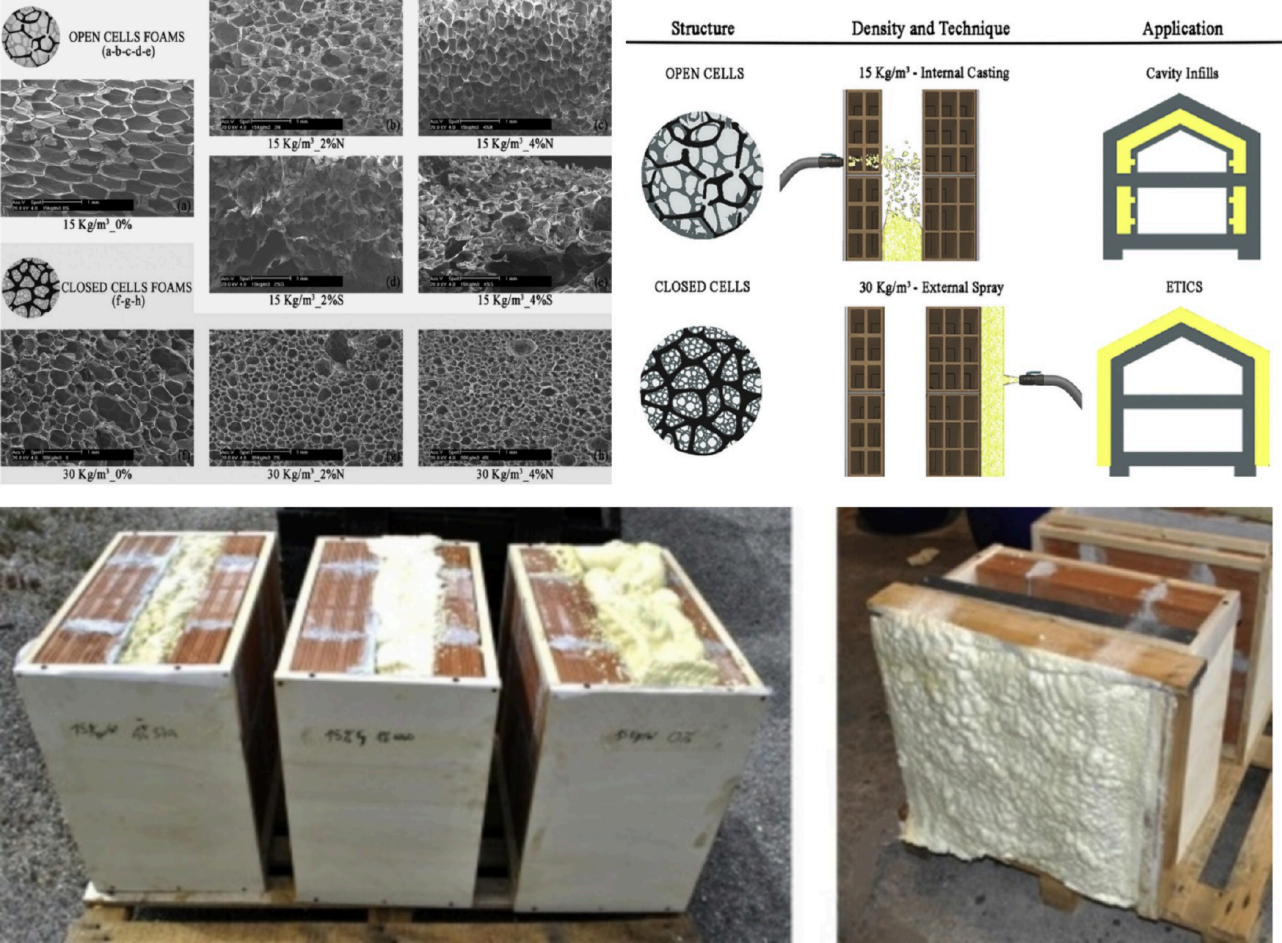
Flexible insulating aerogel for architecture applications. Reproduced with permission from reference.^52^ Copyright 2018 Elsevier Ltd.

Besides, the development of aerogel-based solutions for window systems addresses one of the most critical weak points in building thermal performance. Windows have long been recognized as major contributors to energy loss in buildings, creating an urgent need for innovative materials that can provide both transparency and insulation.^108^ Aerogel technologies offer a promising solution to this challenge by combining excellent thermal properties with optical clarity, opening new possibilities for energy-efficient glazing systems. These materials can significantly reduce heat transfer while maintaining daylighting benefits, creating more comfortable indoor environments with lower energy demands. For example, Zhao et al. selectively extracted sugar cane cellulose from bagasse and construct methyltrimethoxysilane/celluloses composite networks to the fabricate bagasse aerogels.^109^ The hybrid aerogel film exhibited superior flexibility (>15% elastic deformation under 0.02 MPa), high transparency (>80% visible light transmittance), and enhanced thermal insulation (thermal conductivity = 0.0158 W/mK). Additionally, the cost of the 3 mm thick hybrid aerogel film is lower than most reported cellulose aerogels, making it suitable for applications in modern glass buildings.

In the construction industry, driven by increasing emphasis on energy efficiency and environmental protection, flexible aerogels are progressively replacing traditional insulation materials. They are applied in building walls, roofs, floors, and window systems. Aerogels improve overall building energy efficiency and contribute significantly to energy savings and reduced carbon emissions. The flexibility and ease of installation of these materials further promote their widespread use in the construction industry.

### 3.3 Thermal Insulating Applications of Flexible Aerogels in Battery

With the development of electric vehicles and renewable energy, the importance of battery technology has become increasingly prominent. Lithium-ion batteries are favored in the consumer electronics market due to their high energy density, efficiency, and long lifespan.^110^ However, these high-energy-density batteries are more prone to thermal runaway, which can lead to catastrophic hazards due to thermal propagation between cells.^111^ Separators are crucial components in lithium-ion batteries as they isolate high-energy electrodes to prevent internal short circuits and block the spread of thermal runaway from a failing cell to neighboring cells, ensuring battery safety. Flexible thermal insulation aerogels can be used as separators within battery packs, effectively isolating high-temperature regions between modules to prevent thermal runaway. Additionally, the lightweight nature of aerogel materials will not significantly increase the overall weight of the battery pack, preserving the range of electric vehicles. Beyond their thermal insulation, flexibility is particularly critical for battery applications. Aerogels used as separators, coatings, or thermal barriers must tolerate mechanical deformation, assembly stresses, and operational vibrations without cracking. Flexible aerogels can also buffer structural mismatches within modules, absorb mechanical impacts, and accommodate dimensional changes during operation. This adaptability ensures that the insulation layer remains intact, thereby enhancing both the thermal management and the mechanical reliability of the entire battery system.

The application of flexible thermal insulation aerogels in battery thermal management provides critical support for enhancing battery safety and performance. For instance, Deng et al. developed a polyimide aerogel (PIA) separator with uniform porosity, high-temperature resistance, and excellent electrochemical performance using a simple sol–gel method, with its synthesis process and microstructure illustrated in Figure 9a.^53^ The resulting PIA separator exhibited high porosity and flexibility, superior electrolyte wettability, and excellent thermal stability. These outstanding properties enabled the PIA separator to be applied in lithium-ion batteries, demonstrating superior long-term cycling performance and high-temperature stability. Moreover, the PIA separator supported stable cycling of the lithium metal anode at an area capacity of 1 mAh cm^–2^ and effectively suppressed dendritic lithium growth, enhancing the thermal safety of the battery. Liu et al. successfully developed a silica-polyimide aerogel composite separator by incorporating hydrophobic silica with unique functional groups into PI aerogels, as shown in Figure 9b.^112^ The fabricated aerogel separator featured a highly cross-linked 3D nanoporous network, low thermal shrinkage (<4% at 200 °C), high thermal stability (peak temperature of 670 °C), and an oxygen index (>45%). Additionally, the introduction of hydrophobic functional groups reduced polarization in lithium-ion batteries, thereby enhancing power performance and cycling stability.

**Figure 9:**
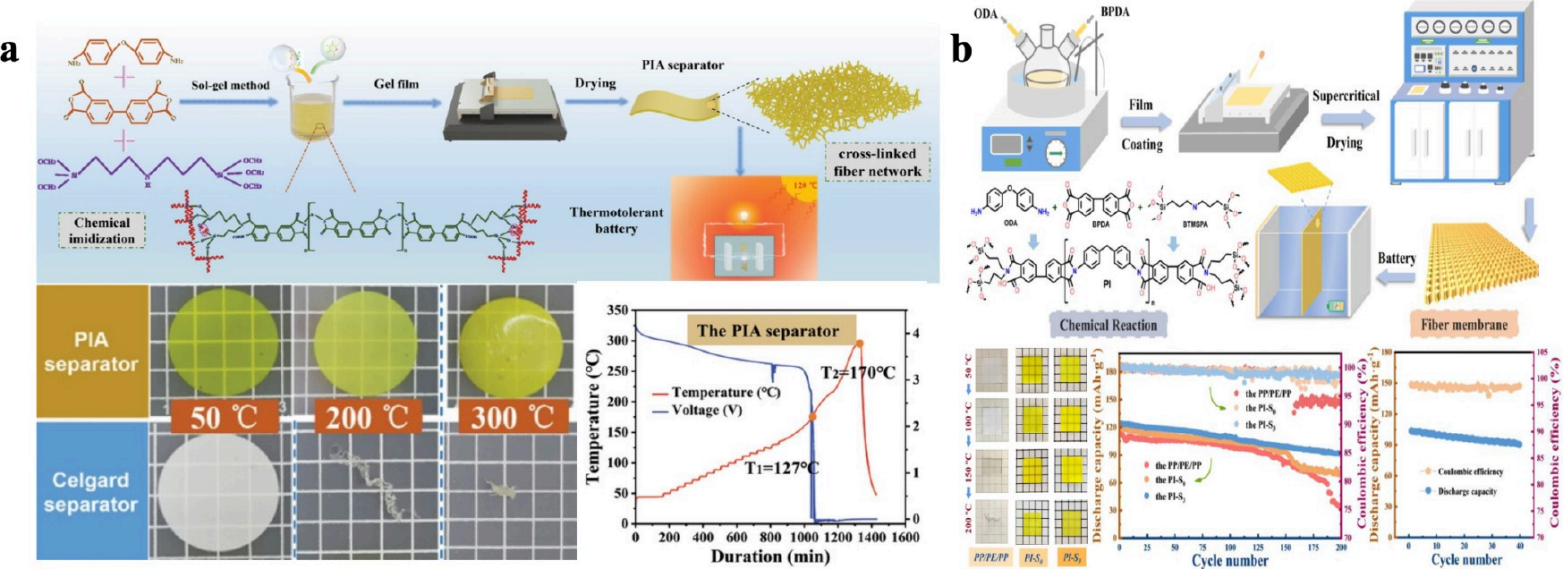
Flexible insulating aerogel for battery applications. (a) Flexible insulating aerogel for lithium-ion batteries applications. Reproduced with permission from reference.^53^ Copyright 2021 Wiley-VCH GmbH. (b) Thermotolerant and flexible silica/PI aerogel composite separator for lithium-ion batteries. Reproduced with permission from reference.^112^ Copyright 2024 Elsevier B.V.

In the battery domain, flexible thermal insulation aerogels can contribute significantly to the thermal management and cooling systems of lithium batteries. Their excellent insulation properties and lightweight characteristics help protect batteries, prevent thermal runaway, and provide reliable support for electric vehicles, aerospace batteries, and other high-performance battery applications.

## 4 Conclusion and Future Outlook

In summary, flexible thermal insulation aerogels, as an innovative functional material, are demonstrating substantial potential across various fields due to their exceptional thermal insulation properties, lightweight nature, flexibility, and diverse applications. The existing fabrication methods mainly include directional freeze-drying, phase separation, 3D printing, and aerogel fibers/fabrics. From aerospace to construction and battery thermal management, flexible aerogels address challenges that traditional materials often struggle with, bringing significant advancements to these domains.

Despite the significant progress achieved with flexible aerogels, several challenges remain. For instance, the relatively high production costs of flexible aerogels limit their adoption in cost-sensitive applications. Additionally, improvements in mechanical properties such as tensile strength and durability are needed to meet more demanding environmental conditions. Future advancements in production techniques and the development of new aerogel materials are expected to broaden the applications of flexible aerogels and enhance their market competitiveness. Looking forward, tentative synthetic routes may involve bioinspired design strategies to optimize the coupling of structural robustness and thermal insulation, providing aerogels with adaptability similar to natural tissues. In addition, constructing hierarchical and hybrid architectures at multiple scales can effectively integrate different components or structures, enabling synergistic improvements in thermal, mechanical, and multifunctional properties. Furthermore, the advancement of scalable and sustainable green fabrication processes—such as ambient-pressure drying, additive manufacturing, and biomass-derived precursors—will be critical to reducing cost barriers and promoting industrial-scale applications. With ongoing technological advancements and increasing market demand, flexible aerogels are poised to leverage their unique advantages in a wider range of applications, contributing significantly to a more efficient and sustainable future.
